# Labia Majora Lipofilling During Body Contouring: Complications

**DOI:** 10.1007/s00266-026-05920-1

**Published:** 2026-06-01

**Authors:** Ahmed A. El Danaf

**Affiliations:** Plastic and maxillofacial surgery department, Mataria Teaching Hospital, General Organization of Teaching Hospitals and Institutes (GOTHI), 139-A Tahrir Street, Dokki, Cairo, 12311 Egypt

**Keywords:** Labia fat graft, Thigh-to-majora flap, Monspexy, Labiaplasty, Tummy tuck, Genital contouring, Adipo-fascial flaps

## Abstract

**Background:**

The increasing requests for enhancement of labia majora during body outline reshaping surgical procedures have garnered special attention. This study focuses on the complications associated with such combined procedures and introduces the thigh-to-labium adipo-fascial flap technique.

**Material and Methods:**

The flaccidity of the labia majora was addressed with autologous fat grafts in 26 patients during abdominoplasty and with adipo-fascial flap from the medial thigh in two patients during thigh lift. The procedure was performed concurrently with mons tacking in ten cases and with edge labiaplasty in four cases. Additionally; a concomitant mammoplasty was performed in 18 cases. Technical details and complications are presented.

**Results:**

The follow-up period for 16 patients ranged from two months to several years. Six patients experienced complications: three (10.7%) related to the tummy tuck, two (8%) to the labial fat grafting, and one the thigh-to-labium flaps. A life-threatening respiratory event in one case was due to excessively tight placation of abdominal wall. A contact burn of the insensate distal abdominal flap was reported in one case. Recurrence of mons ptosis required re-tacking in another. Resorption of over half the volume of the grafted fat necessitated revision in two patients. A labium majus lost prominence in the early postoperative days due to regression of its filling flap from forced evacuative squeezing manipulations of a developed hematoma.

**Conclusions:**

Combining labia majora lipofilling genital and body contouring surgeries may lead to serious complications. Labial fat grafts may experience resorption, while the thigh-to-labium adipo-fascial flap can durably maintain the achieved fullness.

**Level of Evidence III:**

This journal requires that authors assign a level of evidence to each article. For a full description of these Evidence-Based Medicine ratings, please refer to the Table of Contents or the online Instructions to Authors  www.springer.com/00266.

## Introduction

Losing fat from the labia majora can disrupt the youthful appearance of the external genitalia. Many women feel self-conscious about the appearance of their labia majora and request surgical correction [1]. Atrophic majora and sagging mons may negatively impact a woman’s self-confidence. Massive weight loss (MWL), menopausal estrogen deficiency, and aging are common rationales for labia majora flaccidity.

Autologous fat grafting and hyaluronic acid (HA) injections are the prevailing techniques for increasing labia majora volume. A flaccid labium may require approximately sixty milliliters of fat to flourish. The required amount of HA can be highly expensive and may only last for a few months. Filling a flaccid labium with a flap has been reported in a few published articles [2, 3]. The durability of flaps, in general, is more reliable than the long-term outcome of grafts; revisions after fat graft resorption are not infrequent.

The current increasing requests for body contouring trends have led to a rise in demand for associated esthetic genital surgeries. Monsplasty in older patients is a common phase during abdominoplasty. Mons tacking sutures reduce mons ptosis and prevent excessive mons elevation by the upward pulled abdominoplasty flap. It is recommended that the first midline stitch be placed several centimeters away from the anterior labial commissure to achieve a natural look [4]. Mons cutaneous redundancy and sagging often require direct excision and lifting [5, 6] which can stretch the upper parts of labia majora skin [7]. Fat grafting employs the waist product of liposuction which is a frequently performed maneuver during body contouring surgery. Abdominal wall repair particularly after MWL often includes monspexy [8–10].

Despite the increasing popularity of labia majora contouring in recent decades, there is a lack of scientific publications on the topic [11–14]. This article aims to discuss the complications of combining majora lipofilling with body contouring procedures and provide information on the thigh-to-labium adipo-fascial flap technique.

## Material and Methods

This is a retrospective study of surgeries carried out between 2008 and 2022. Data were directly collected over the years. Approvals were given by the ethical boards of the private hospitals where operations were performed. Consent forms including permission for potential use of images for educational purposes were signed by the patients.

Twenty-eight patients who were assigned to undergo abdominal liposuction and/or abdominoplasty requested labia majora enhancement. The service was offered in the private accredited facilities; most, if not all, patients seemed belonging to a high socioeconomic standard. They were seeking to rejuvenate their private areas and enhance their self-confidence.

**Fig. 1:**
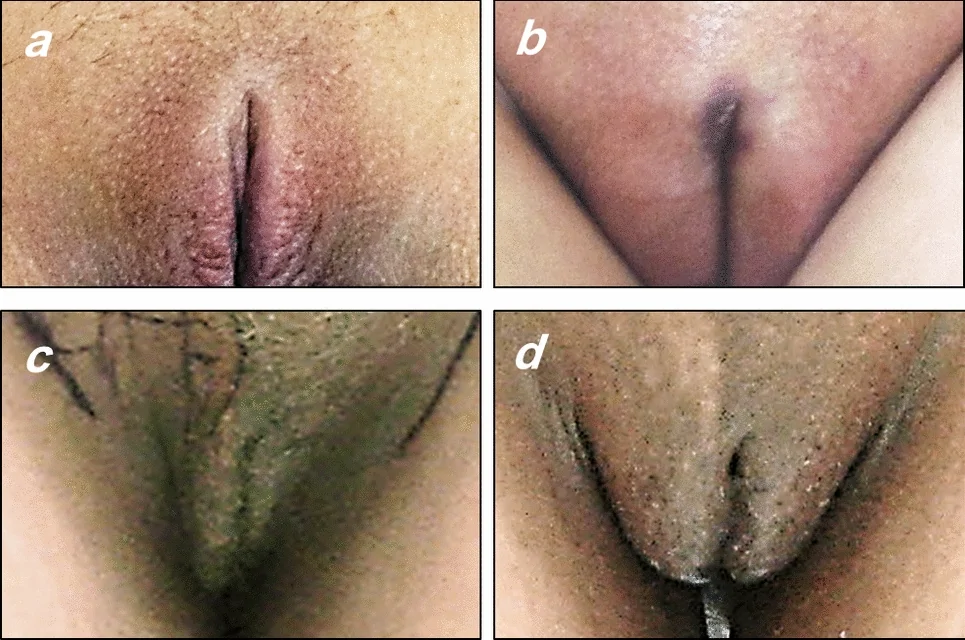
Fat graft inflating thin labia majora and stretching its wrinkles: Upper row: Preoperative **a** and 5-day postoperative **b** photographs of 55-ml labial fat graft per labium majus in one patient; Lower row: Preoperative **c** and 8-day postoperative **d** photographs of 50-ml fat graft per labium majus and 120 ml to the mons in another postmenopausal elder patient filling a posttraumatic right-side defective contour

**Fig. 2:**
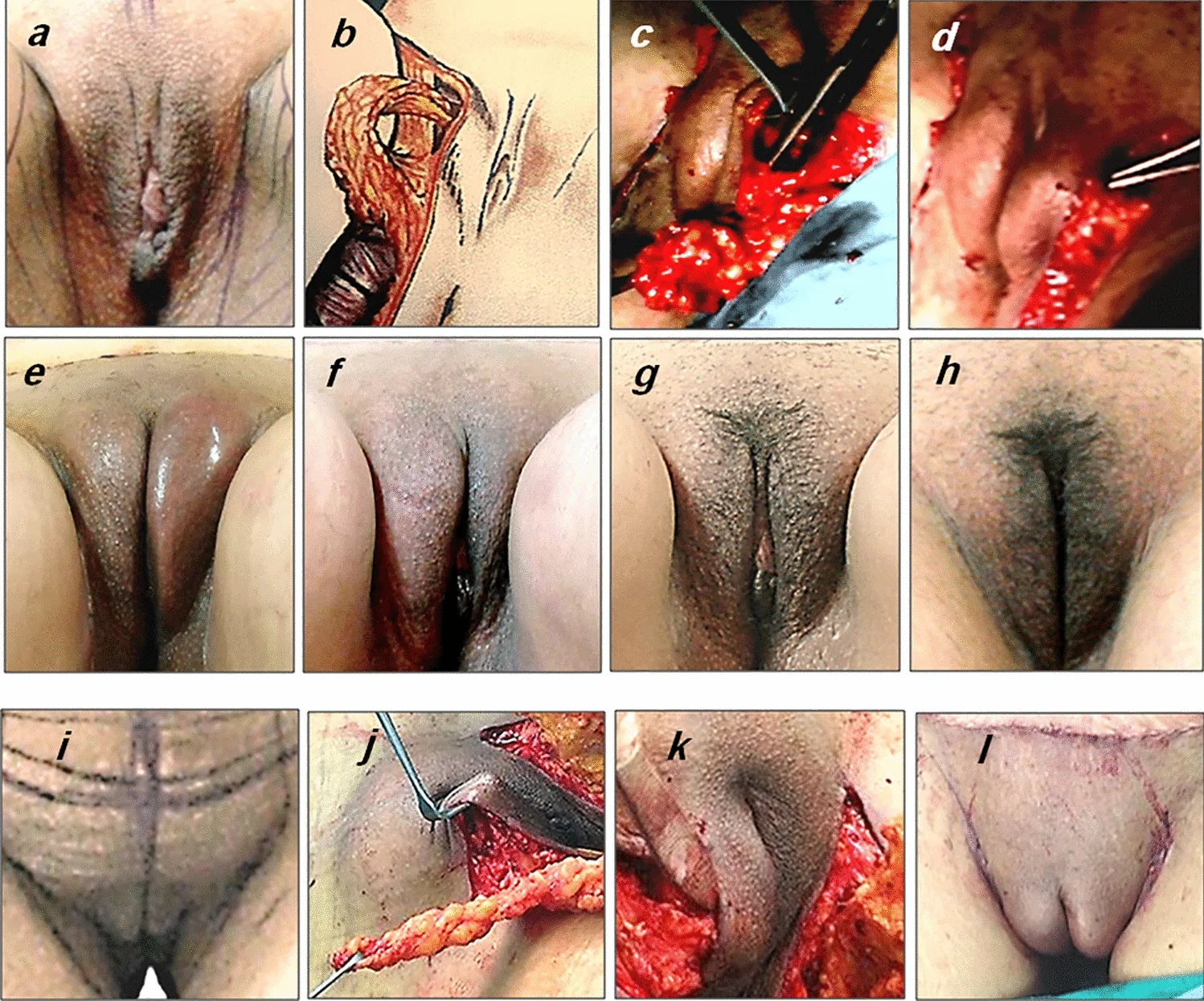
Labial lipofilling by a thigh adipo-fascial pedicle flap: Two patients underwent labial augmentation combined with thigh lift, abdominoplasty, and mons tacking. First case (Upper and middle rows): Preoperative photograph **a**, flap inset drawing **b**, intraoperative photographs displaying the right labium already filled by the flap and the left labium receiving the flap through an upper small fascial window **c**, **d**, early postoperative photograph showing left labium majus puffed with a developed hematoma **e**, few weeks after hematoma evacuation photograph **f**, and one-year postoperative photograph showing the left labium after regaining volume with 60-ml fat grafting, before **g** and after **h** later labia minora minimal peripheral trimming. Second case (Lower row): Preoperative photograph (**i**), intraoperative photographs **j**, **k** showing the flap and the entry window of the recipient labial tunnel and the immediate postoperative (**l**) photo. Fig. pieces 2**a**–**f** were previously published [2]

In 26 abdominoplasty cases, a thigh lift procedure was not requested by the patient and the labial augmentation was achieved using fat grafts (Fig. 1). Thigh adipo-fascial flaps were accepted and performed in two patients who requested a combined thigh lift along with tummy tuck (Fig. 2). The patients’ ages ranged from 20 to 63 years old with a mean age of 42. Twelve patients were postmenopausal, and three were under 30 years old and had a history of MWL. The operative time ranged from 3 to 8 hours and prophylactic antibiotics, anticoagulants, and intraoperative leg pneumatic cuffs were routinely used.

### Labia Majora Augmentation Operative Techniques

Fat grafts:

Autologous harvested fat was decanted and transferred without washing, centrifuging, or adding platelet-rich plasma. The faint yellow oily and reddish serum liquids above and below the pure yellow fat were discarded from syringes before grafting. A blunt 2-mm cannula was used for bilateral labial fat injections through a single large-gauge needling entry point on the mons midline; additional entry points were rarely needed. The new fat was inserted into the labium in multiple thread-like rows primarily filling the medial half of the labium. The labial border between the medial and outer aspects was held between the thumb and index finger to precisely guide the fat insertion as the cannula was slowly withdrawn. The grafted fat varied between 25 ml and 80 ml per labium with an average of 57 ml including approximately 10 to 20% overcorrection to account for potential partial fat absorption. The anterior half of the labium received nearly double the amount of fat compared to the posterior half to end with a natural shape. Some fat was also injected into the nearby mons areas to address any prominence differences between the labia majora and the mons. Gentle massage with fingertips evenly distributed the fat and possibly improved graft survival rates by increasing the donor-recipient surface area. Any remaining depressions were filled in with additional fat transfers. Symmetry was carefully checked, and any minimal differences were addressed.

Thigh-to-labium adipo-fascial flap:

The adipo-fascial flap technique was applied in only two patients whose labia majora augmentation was combined with a thigh lift to make use of the disposable thigh tissue. The upper medial thigh fascia with its fat tissue was elevated based on a superior pedicle and transposed to fill the adjacent labium. The bed and lateral border of the proximal third of the flap were left intact without incision and without dissection of their attachments to preserve and maintain the flap blood supply. The anterior branch of the obturator artery, the superficial and deep external pudendal arteries, and the medial circumflex femoral arteries anatomically contributed to the flap vascularity. The length of the elevated flap paddle was triple the length of the labium so as to ensure its sufficiency. A couple of centimeters opening window was made on the medial upper border of the flap to create a labial entry. A recipient pocket was prepared along the full length of the medial border of the labium. The flap was then passed subcutaneously to fill the labial blind tunnel, turning it inside out in a manipulative manner. The flap should have been better secured in its new position by posterior labial trans-cutaneous absorbable stitches to avoid any accidental dislodging event.

### Combined Esthetic Surgeries (Table 1)

**Table 1:** Patients age, labial fat graft amount, and accompanying genital and body procedures

*N* ^o^	Patient age in years	Labium lipofilling	Combined genitalia procedures	Abdominal procedures	Other combined body contouring procedures	Complications	Follow-up in months
1	20	80 ml	Mons lift	Lipo-abdominoplasty without recti sheath plication	Breast implant 375cc sub-pectoral insertion	Lower abdominal contact burn by hot bag	2–18
2	54	70 ml		Lipo-abdominoplasty with umbilical hernia	Mastopexy Buttocks fat graft 1.2 liters / side		>18
3	43	50 ml x 3 (=150 ml in three sessions)		Lipo-abdominoplasty	Breast reduction 310 g / side Buttocks fat graft 550 ml / side (600 ml in the first revision and 350 in the second)	Revision of labial and gluteal fat grafting in two occasions	2–18
4	51	65 ml			Breasts fat graft 450 ml / side Thighs lift Facial fat graft 15 ml / side		
5	46	50 ml			Breasts fat graft 145 ml / side Hips fat graft 150 ml / side		
6	38	40 ml			Breasts fat graft 390 ml right side & 370 left side Inverted nipples correction Back & thighs 2 liters, and sub-mental 10 ml liposuction		< 2
7	29	60 ml			Brachioplasty		
8	28	Adipo-fascial thigh flaps			Mastopexy combined with breast implant insertion Buttocks lift and auto-augmentation by adipo-fascial posterior thigh flaps		
9	47	Adipo-fascial thigh flaps and revision by left labium fat grafting and later limited labiaplasty		Mini-abdominoplasty	Thighs lift Abdominal & thigh 7 liters liposuction	Unilateral labial hematoma	2–18
10	55	60 ml	Mons lift 150 ml liposuction	Lipo-abdominoplasty			
11	63	50 ml	Mons 120 ml fat graft filling a right scared depression	Abdominal liposuction 3.3 liters	Buttocks & hips fat graft 1.6 liter / side Back 2 liters liposuction		
12	47	55 ml (after removal of old solid permanent filler), and 60 ml 2 years later	Labiaplasty Mons 32 ml fat graft	Lipo-abdominoplasty	Breast implants exchange	Revision of labial fat grafting with 60 ml 2 years after surgery	>18
13	30	50 ml (and repeated a year later by 40 ml)	Labiaplasty	Abdominal liposuction	Breasts 400 cc implants exchanged by 320 cc and 240 ml fat grafts, and mastopexy to correct riding NAC Secondary buttocks lift	Revision of labial fat grafting with 40 ml one year after surgery	2–18
14	27	75ml			Buttocks fat graft 900 ml /side Trunk 5 liters liposuction		< 2
15	24	45 ml		Lipo-abdominoplasty	Buttocks lift Iliac regions liposuction		
16	38	75 ml			Breasts fat graft 700 ml / side		
17	42	75 ml			Buttocks lift with fat graft 950 ml on right side & 800 ml on the left		
18	33	50 ml			Buttocks fat graft 350 ml / side		2–18
19	59	25 ml			Mastopexy Fat graft to hips supra-trochanteric areas 350 ml / side		
20	31	75 ml		Abdominal above 2 liters liposuction	Breasts fat graft 320 ml / side		< 2
21	48	60 ml			Breasts fat graft 500 ml / side Buttocks fat graft 900 ml / side Brachioplasty		
22	49	50 ml			Breasts fat graft 300 ml / side		
23	43	50 ml			Buttocks 750 ml and hip supra-trochanteric area 350 ml fat grafts Back and arms 3 liters liposuction		
24	44	50 ml			Breast fat graft 380 ml / side Buttocks auto- augmentation with adipo-fascial flaps		2–18
25	42	50 ml			Buttocks & hips fat graft 300 / side		
26	52	35 ml			Breasts fat graft 400 ml / side Trunk and thighs 7 liters liposuction		
27	46	60 ml			Breasts fat graft 300 ml / side Hips 500 ml & face 17 ml fat grafts		
28	37	50 ml		Secondary abdominoplasty (fibrosis from previous power-assisted liposuction)	Breasts fat graft 300 ml / side Hips trochanteric areas fat graft 350 ml / side

**Fig. 3:**
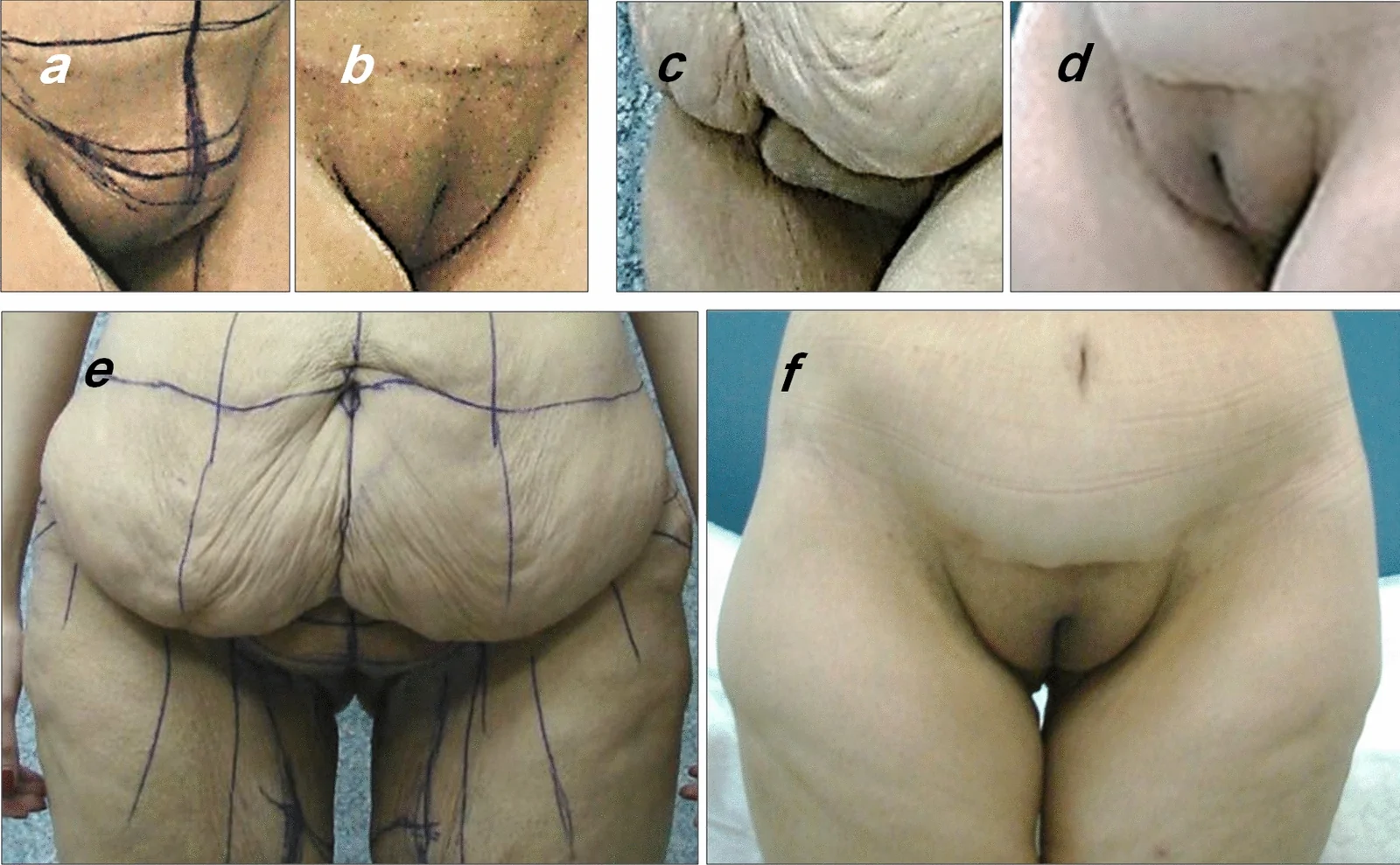
Mons lift enhancing the labial appearance: Preoperative **a** and 2-month postoperative **b** photographs of monspexy combined with mons 200-ml liposuction in a patient who had previous abdominoplasty with missed mons tacking to Scarpa’s fascia and rectal sheath. Preoperative **c** and **e** and 3-year postoperative **d** and **f** photographs showing mons lift stretching up labia majora redundancy in another patient who had history of massive weight loss

**Fig. 4:**
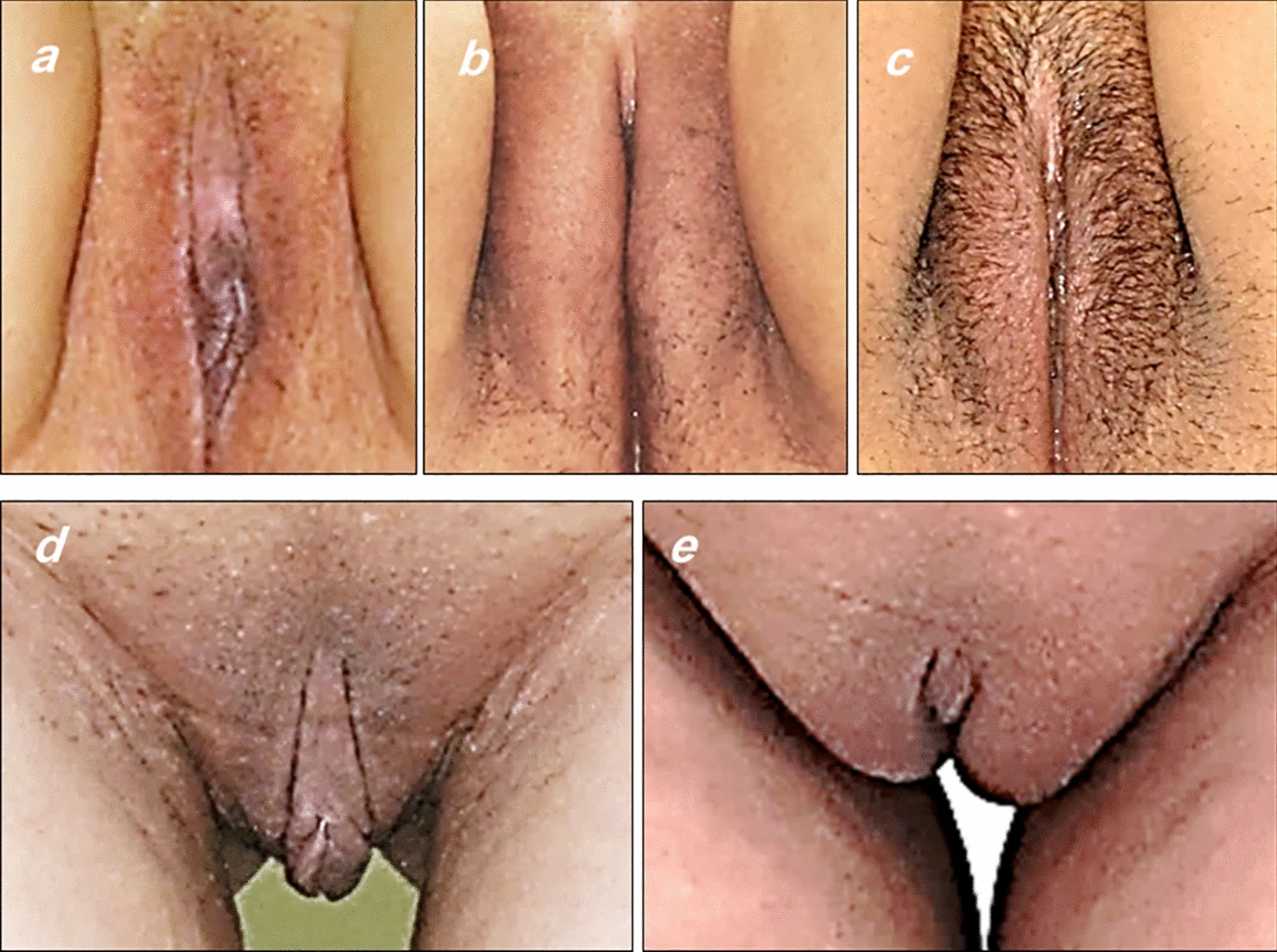
Labiaplasty: A patient underwent 50-ml fat grafting per labium few months after removal of a couple ml of solid tiny beads permanent filler of unknown material, pre **a**, 6-month **b**, and 3-year postoperative **c** photographs. Another patient who had 55-ml fat grafting to both labia majora and mons and the operation was revised a couple of years later by further majora 60-ml and mons 32-ml fat grafting in combination with an edge labiaplasty procedure; preoperative **d** and 8-month postoperative **e** photographs

- External genital procedures:
  - Monspexy was simultaneously performed in ten patients (Fig. 3). Mons ptosis, which partially or completely, hides the external genitalia and is rated as a Pittsburgh scale 2 or 3, respectively, was lifted through an upper inverted trapezoidal excision and tacking of the deep sub-dermal fatty content to Scarpa’s fascia and anterior sheath of the rectus abdominis muscles. When the original location of the umbilicus was high, equidistant from the sternal xiphoid and the pubis, balancing the size of the excised upper sector of the mons with the extent of abdominal flap resection was particularly challenging in order to avoid an undesirable inverted-T abdominoplasty scar. One patient with a history of MWL who underwent monspexy had large labia majora but she did not request reduction (Fig. 3d and f).
  - Trimming of protruding labia minora was performed in four patients, with one patient also receiving a 32-ml fat grafting to the mons (Fig. 4).
  - Mons grafting with 120 ml of fat was performed in one patient to address an extensive old posttraumatic depressed scar (Fig. 1c).
  - A bulging mons in one patient was reduced through 150-ml liposuction prior to the majora fat grafting.
- Body contouring procedures:
  - The abdomen was interested in all patients; 15 lipo-abdominoplasty, 2 abdominoplasty, and 11 abdominal liposuction procedures. Preoperative abdominal wall ultrasonic examination excluded any hidden hernias. In one patient, undermining the abdominal flap required the use of a knife because of extensive tough fibrosis attributed to a previous power-assisted liposuction. A young patient with history of MWL had strong abdominal muscles that did not necessitate the midline approximation of the rectus sheathes.
  - Breasts were involved in 18 cases, buttocks in 13, thighs in 2, and arms in 2.

## Results

Labia majora lipofilling effectively rejuvenated the vulva and almost all patients were satisfied. Patient satisfaction evaluation was based only on clinical impression; a structured questionnaire or a visual analog scale would provide a more reliable systematic assessment. The operative time for the genital procedures did not exceed an hour. Fourteen patients who underwent a labial fat grafting procedure were followed for 2 to 18 months, two patients, one with flap, for over 18 months, and twelve patients, one with the flap, for less than 2 months.

### Complications

As the series in this study involved combined diverse procedures and variable fat graft volumes, the reported complications could not determine specific rates or constant etiologies. Six patients (21%) had complications. Three out of 28 (10.7%) were related to the tummy tuck, and two out of 26 (8%) to the labial fat grafting. One out of the four thigh-to-labium flaps was accidentally forced backup. Complications were classified according to the time of onset and to the surgically addressed body region as follows:

- Early postoperative complications:
  - Labia majora lipofilling relevant difficulties included:
    - Temporary bruises, edema, surface irregularities, and asymmetries were minimal and inconsistent. Infections were absent in this series of patients.
    - Clinical examinations, few hours after surgery and in the early follow-up visits, checked the genital region to exclude any potentially developed hematoma especially with the use of prophylactic anticoagulants. A labium majus filled with adipo-fascial flap developed a hematoma. It was evacuated on the 8^th^ postoperative day. The distal half of the flap was possibly pushed upwards during mandatory manual squeezing of the hematoma; fixation of the flap tip by a few stitches through a small incision at the labial posterior extent would have prevented such accidental mishaps. The resulting contour defect was compensated with fat grafting few weeks later.
  - Accompanying abdominoplasty relevant complications involved:
    - Excessive abdominal tightness caused by exaggerated midline approximating of the anterior rectal sheaths prompted the rapid transfer of a recovering patient from the ICU to the OR. Surgical removal of the superficial two out of three rows of stitches released the strained respiration. An alert anesthesiologist should immediately notify the surgeons if the patient’s end-expiratory pressure rises by more than 5 mmHg during abdominoplasty.
    - A lower abdominal temporary hypoesthesia occurred in a 20-year-old patient after abdominoplasty. This was due to a contact superficial second-degree burn caused by the patient using hot bags to relieve menstruation pain. The lesion healed within a couple of weeks without the need for surgical intervention. Patients should always be informed about potential side effects of surgery.
- Late postoperative complications:
  - Palpable nodular formations and fibrosis in the grafted labia majora were not present. The overcorrection of fat grafting was appreciated by most of the patients.
  - Revision of fat grafting was indicated when over half the gained volume was lost due to fat resorption and was based on the patient-driven esthetic concerns. Fat graft to labia majora had to be repeated with 60 ml 2 years in one patient with initial grafting of 55 ml. Revision was performed twice in another patient with 50-ml fat graft per session at few months intervals. Resorption of the grafted labial fat was evaluated depending on the clinical examinations and photographic comparison during the follow-up visits; a volumetric measurement would yield reliable precise figures.
  - Recurrence of mons ptosis few months after inadequate surgical tacking in one patient was revised with more than five stronger non-absorbable stitches.

## Discussion

The size of the labium majus mainly depends on its fat content. It is commonly wider and taller anteriorly than posteriorly, measuring an average length of 7.5 cm and width of 2.5 cm. The labia majora, along with the mons veneris, make up a single cosmetic unit anatomically referred to as the anterior "level" or "first ring" of the vulva. They conceal the pinkish labia minora, clitoris hood, and vestibule [15].

Autologous fat grafting may be the preferred method for adding volume to atrophic labia majora, restoring a youthful appearance. It increases the patient satisfaction rate when combined with different esthetic surgeries. Fat grafting of 20 to 120 ml per a flaccid labium majus can adequately rejuvenate it [3]. Labial prominence in continuity with mons fullness can protect against dryness and enhance enthusiasm [16]. Labia majora lipofilling allows for less tissue reduction during labiaplasty, with a high rate of patient satisfaction [17]. Many plastic surgeons tend to avoid labia majora reduction [10], as it could potentially decrease volume without effectively addressing skin wrinkles. Surgical excision of labia majora skin may expose the internal introitus, desiccate the vagina, and induce visible scars. Local estradiol therapy can help with vaginal dryness and dyspareunia [18], but it does not address labia majora attenuation, flatness, and skin shrinkage. Fillers such as hyaluronic acid and calcium hydroxyapatite have been injected to add volume to the labia majora [19]; yet the large volume needed to fill labia majora may be expensive in view of its few month benefits.

Cases of pulmonary embolism have been reported after fat and filler injections around the vagina [20] and into the buttocks [21]. Implementation of precautions during gluteal fat transplantation should enhance safety [22, 23]. The fat embolism syndrome with the typical triple sign of hypoxia, reduced consciousness, and skin petechial rash usually starts during the first 3 postoperative days [24]. A mortality rate of 34.3 % and sequels in 88.6 % of survived patients were recently reported [23]. Combining esthetic surgical procedures, as long as the operative time is not expected to exceed 8 hours, may allow harmonious improvement of the outlines of different body parts. A comparative study showed that adding abdominoplasty to mammoplasty in one session did not increase complications [25]. Surgeries after MWL are preferably combined in no more than two operations [26].

The small sample size in this study did not encourage building a comparative analysis between labial fat grafting and thigh-to-labium adipo-fascial flap. The work involved more than one surgical procedure and variable follow-up durations.

## Conclusion

Labia majora lipofilling combined with esthetic surgical procedures may lead to serious complications. While labial fat grafts may require overcorrection or revisions, thigh-to-labium adipo-fascial flap can sturdily preserve the enhanced labial volume.
